# Hemodynamic monitoring in the era of digital health

**DOI:** 10.1186/s13613-016-0119-7

**Published:** 2016-02-17

**Authors:** Frederic Michard

**Affiliations:** MiCo, Denens, Switzerland

**Keywords:** Hemodynamic monitoring, Digital health, Imaging, Graphical displays, Sensor, Wearable technology, Connectivity, Internet of things, Mobile health, Home monitoring

## Abstract

Digital innovations are changing medicine, and hemodynamic monitoring will not be an exception. Five to ten years from now, we can envision a world where clinicians will learn hemodynamics with simulators and serious games, will monitor patients with wearable or implantable sensors in the hospital and after discharge, will use medical devices able to communicate and integrate the historical, clinical, physiologic and biological information necessary to predict adverse events, propose the most rationale therapy and ensure it is delivered properly. Considerable intellectual and financial investments are currently made to ensure some of these new ideas and products soon become a reality.

Digital innovations are changing the world at the speed of sound. Examples are multiple in our daily life: internet, social networking, smart phones, tablets and GPS, just to mention a few. They are also changing medicine [1, 2], and hemodynamic monitoring will not be an exception. In this viewpoint article, using published and public information, I will describe technological and digital innovations which will likely transform hemodynamic monitoring within the next 5–10 years. For sake of clarity, although they are linked, I have separated innovations into three categories: imaging, sensors and connectivity.

## Imaging

**Fig. 1:**
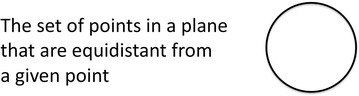
An example of the “picture superiority effect.” A drawing is worth many words to describe a simple circle

Monitoring is not treating but providing physiologic information to clinicians. This information is used to better characterize and treat disease states. The way the information is delivered is pivotal to making a difference. This is demonstrated using aviation as an example, where safety and efficiency have always been top priorities. The way the information is delivered in the cockpit has changed dramatically over time. Graphical displays have replaced knobs and dials to give pilots an integrated visualization of flying conditions. One of the main goals of visualization is to make possible the absorption of large amounts of data quickly [3–5]. The brain processes pictures all at once, but processes text in a linear fashion (Fig. 1). Pictures allow the immediate recognition of patterns and trends [3–5].

### Metaphor and target screens

**Fig. 2:**
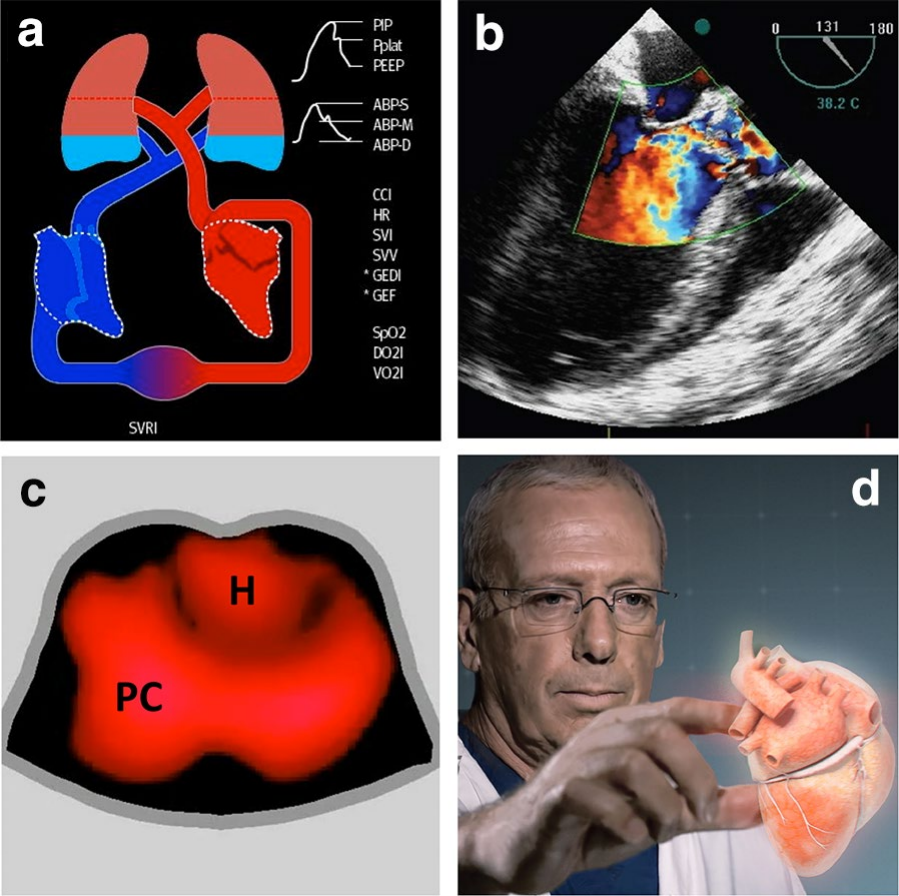
Examples of bedside digital methods useful to “look” at the cardiopulmonary circulation. **a** Graphical display, HemoSight screen from Mindray with permission; **b** echocardiography Doppler, **c** electrical impedance tomography, *H* heart, *PC* pulmonary circulation, from Dixtal with permission; **d** digital holography, downloaded from Realviewimaging.com

**Fig. 3:**
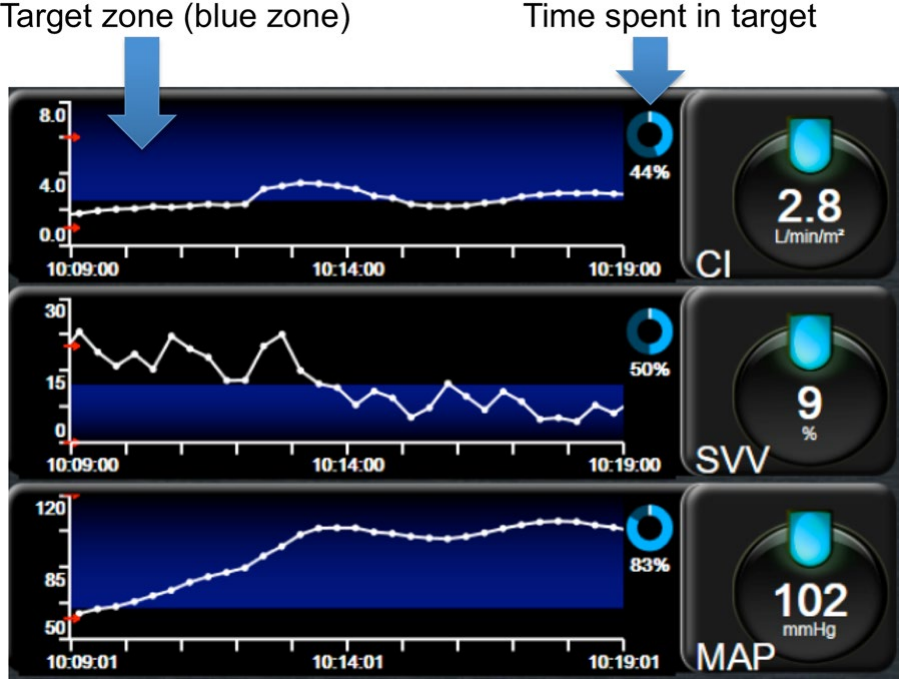
Example of target screen. Screen designed for perioperative goal-directed therapy. In this example, goals are to maintain cardiac index (CI) >2.5 l/min/m^2^, stroke volume variation (SVV) <12 % and mean arterial pressure (MAP) >65 mmHg. Visualization of target zones and quantification of time spent in target are an invitation to follow the hemodynamic protocol. From Edwards Lifesciences with permission

There are different ways to make visual the information provided by monitoring tools. The first one is to create graphical displays. Several studies have demonstrated that, when compared to classical numerical displays, graphical displays allow a faster detection of changes in physiologic variables, a more accurate diagnosis and/or a decreased mental workload [6]. Graphical displays can be cartoon-like representations of the human body (Fig. 2). This is the principle of metaphor screens which are, for hemodynamic monitoring, graphical representations of the cardiovascular or cardiorespiratory system [7]. They are useful to put meaning on parameters, particularly for trainees and nurses. Graphical displays can also be abstract representations of hemodynamic parameters. Target screens belong to this category [7]. They are designed to help clinicians chasing one or more targets. They “alarm” practitioners as soon as they deviate from their protocol, and have the potential to improve compliance, a key element of success when goal-directed strategies are followed. They can also be used to quantify how much time is effectively spent in target, a simple way to track and report protocol adherence (Fig. 3).

### Real images

The second method to make visual the information provided by monitoring tools is to use technologies allowing a direct visualization of human anatomy and physiology, the most common example being ultrasound (Fig. 2). It is amazing to see how echo devices have evolved over time, from heavy and big machines to handheld or even pocket devices [8], with now the option to connect an echo probe to a cell phone [9] or to use disposable esophageal probes for continuous monitoring [10]. Technological improvements have been so quick that human skills or lack of training is today the main limitation to the use of ultrasounds by anesthesiologists and intensivists. Electrical impedance tomography (EIT) is another bedside method to see inside the body. Thoracic belts are used to send and record electric signals from dynamic intrathoracic structures such as lungs and cardiac chambers. Images are not anatomical but functional, and EIT is currently used to assess the effects of mechanical ventilation on lung function. In the future, it may also be used to assess heart–lung interactions [11] and lung edema [12], as well as to assess lung perfusion [13], opening the door to the assessment of ventilation/perfusion mismatch, the main cause of arterial hypoxemia in critically ill patients. Finally, EIT has the potential to be used to assess stroke volume and cardiac output and to create cardiovascular images (Fig. 2). Holograms are not a dream anymore since the development of a digital system allowing cardiac surgeons to visualize and manipulate the heart in three dimensions [14]. For anesthesiologists and intensivists, this may also become one day a way to “look” differently at the cardiovascular function (Fig. 2).

**Fig. 4:**
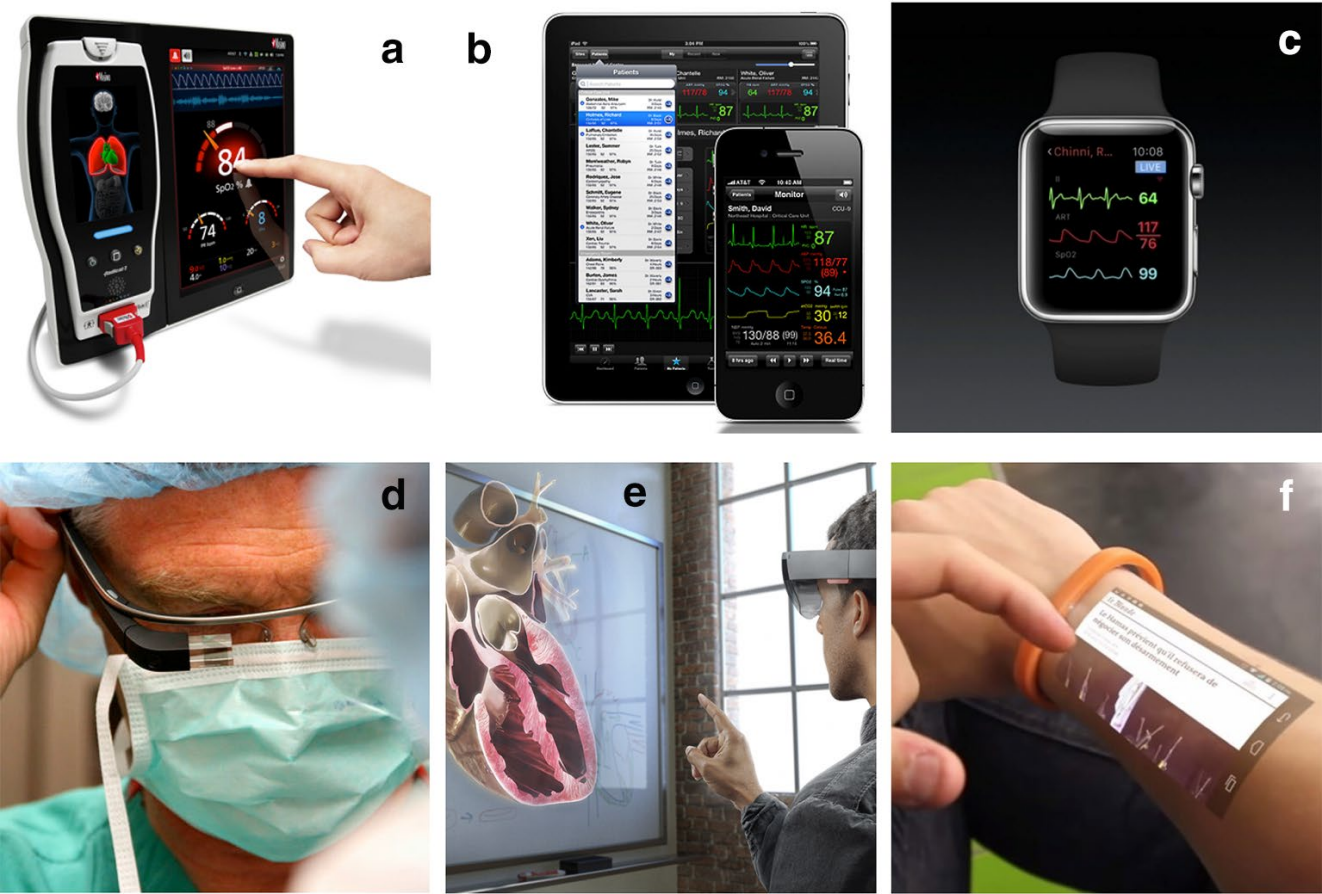
Examples of digital methods to display monitoring information. **a** Bedside monitor, downloaded from Masimo.com; **b** tablet and cell phone, downloaded from Airstrip.com; **c** digital watch, downloaded from Apple.com; **d** connected glasses, downloaded from Google Images; **e** lenses allowing the visualization of images in three dimensions, downloaded from Microsoft.com; **f** laser pico-projection turning skin into a touch screen, downloaded from Cicret.com

Technically speaking, both graphical displays and images can be displayed on computers, tablets and cell phones (Fig. 4). Head-mounted systems, such as glasses or lenses [15], and wearable wrist systems, such as digital watches or laser pico-projectors [16], are also an option for the future (Fig. 4). Whether these alternatives may have any advantage over classical visualization on bedside monitors remains to be determined by care givers.

### Imaging in education

Several studies have shown that the lack of knowledge is a significant limitation to the correct interpretation of hemodynamic variables [17]. Serious games are video games designed to learn and train on serious topics, such as safety and processes. Serious games are increasingly used in industry and hospitals [18]. They increase the use of learning tools (this is fun) and training efficiency (this is simulation) [19]. Advantages of simulation training over traditional education methods include unlimited exposure to rare but complicated clinical events and the absence of risk for patients [20]. Simulation-based training was demonstrated to improve medical knowledge, clinical performance and comfort in procedures. It is also useful to teach teamwork and communication, a key element of quality of care [21]. Therefore, the current development of serious games in health care [18] may greatly benefit hemodynamic monitoring, given the large number of hemodynamic variables and tools, as well as the complexity of cardiovascular physiology.

## Sensors

Over 1 million pulmonary artery catheters are still used every year. However, many alternative monitoring technologies have emerged over the last decades, from esophageal Doppler to bioimpedance tracheal tubes [22], bioreactance skin patches [23] and pulse contour methods. Pulse contour algorithms allow the computation of stroke volume and cardiac output from an arterial blood pressure curve and are constantly evolving [24]. Their reliability mainly depends on the quality of the pressure signal and on changes in vascular tone, as those induced by sepsis, cirrhosis and the use of vasoactive drugs [25, 26]. In this context, their accuracy and precision has been questioned by several studies using thermodilution or echocardiography as reference methods [25, 26]. However, because they allow real-time monitoring, are easy to use, not operator dependent and not affected by electrocautery, pulse contour methods have become in a few years the clinicians’ preferred choice for the hemodynamic management of patients undergoing major noncardiac surgery [27].

The arterial pressure curve can be recorded from an arterial line, but also noninvasively, from finger arteries with the volume clamp method [28], from the radial artery by applanation tonometry [29] and soon from a brachial cuff by hydraulic coupling [30]. The arterial pressure waveform can also be captured from any bedside monitor by a cell phone camera, and hemodynamic parameters can be computed by a downloadable application [31, 32]. This last option has not yet been approved for clinical use, but it illustrates the ability of digital technologies to push the envelope of hemodynamic monitoring practices.

### New pressure sensors

**Fig. 5:**
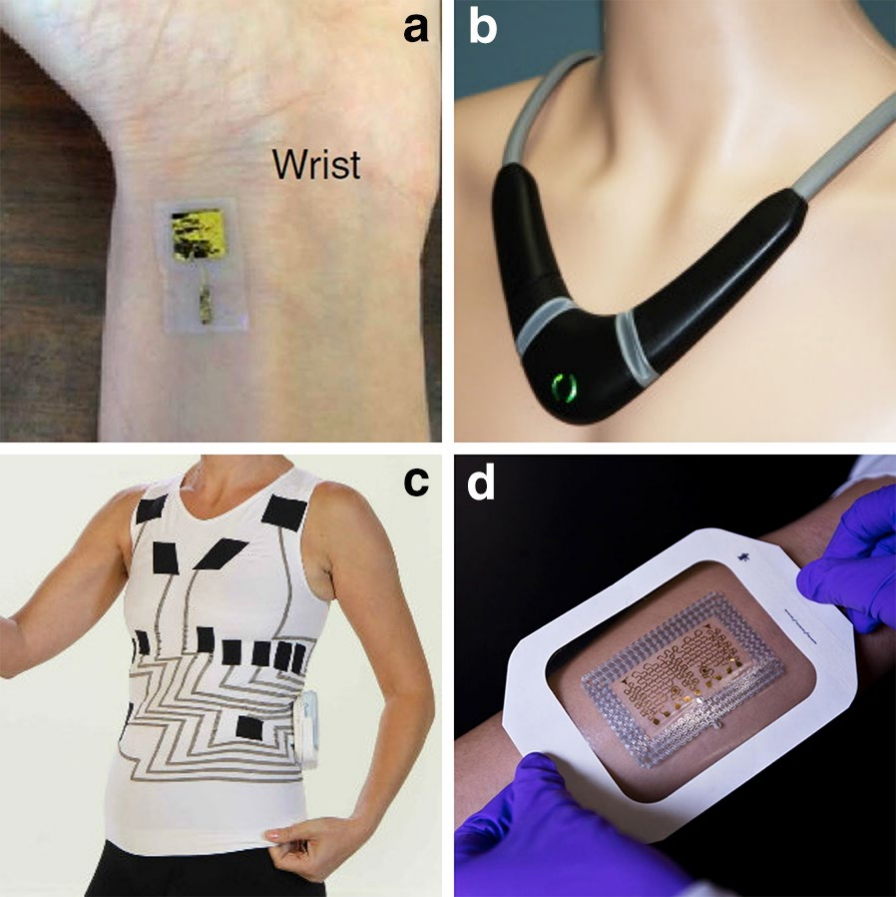
Examples of wearable and wireless sensors. **a** Wireless patch measuring radial blood pressure, from [34] with permission; **b** necklace estimating thoracic fluid content, downloaded from Tosense.com; **c** T-shirt recording 12 leads ECG, downloaded from Personal-healthwatch.com; **d** electronic tattoo, downloaded from engr.utexas.edu

Micro- and nanoelectro mechanical systems (MEMS and NEMS, respectively), biochemical innovations and 3D printers are about to revolution the world of pressure sensors. Thanks to these technological advancements, sensors can be miniaturized. Some are no larger than a Band-Aid or a pinhead [33, 34] and, when placed next to the radial artery or the carotid artery, are able to provide an arterial pressure signal (Fig. 5). Because they have the potential to be cheap (thanks to 3D printers), and because they are tiny, noninvasive and wireless, such sensors may make continuous blood pressure monitoring a reality beyond the operating room and ICUs. This may benefit to many patients, from those in the wards at risk of hemodynamic deterioration—the large EUSOS study [35] showed that most patients who do not survive a surgical stay die in the wards—to outpatients with chronic hypertension. And if carotid artery pressure becomes easy to monitor, pulse contour methods should gain in accuracy, because of the assumptions made by current algorithms when analyzing peripheral pressure waveforms. Miniaturization of sensors also makes possible measurements in situ. Small pressure sensors can now be positioned in the pulmonary artery during a right heart catheterization procedure and transmit on demand pressure measurements to an external unit. Studies have shown that they allow home monitoring of patients with chronic heart failure, early detection of deterioration, early modifications of treatment and a highly significant decrease in hospital readmission [36]. More recently, other mini-sensors have been developed for intracardiac pressure measurements [37]. Implanted during cardiac surgery they will allow atrial or ventricular pressure monitoring after surgery, in the hospital and also from home. Other noninvasive wearable sensors [38, 39] allow an estimation of the intrathoracic fluid content and have potential to early detect a deterioration in left ventricular function and/or fluid overload in patients with chronic heart or kidney failure (Fig. 5).

### Regional perfusion monitoring

A myriad of new technologies is also becoming available to explore regional perfusion: sublingual microcirculation, gastric mucosal perfusion, thumb muscle oxygenation, brain perfusion and oxygenation, just to mention a few. Usability of these tools remains an issue, but some have gained in practicality and reproducibility over the last few years [40]. Of note, a recent consensus of hemodynamic experts [41] considered that the use of these techniques should be limited to research until we can answer the following questions: When should we treat a decrease in regional perfusion (some may be adaptive mechanisms, such as kidney and skin hypoperfusion during hemorrhage), how should we treat them, and does it impact outcome?

### Metabolic monitoring

Metabolic sensors are also evolving. In patients with an arterial catheter or a central venous catheter, in situ measurements of blood gases and electrolytes are now possible [42]. They allow on demand measurements, quick results (no need to send anything to the laboratory), without any blood loss (no blood sample). Electronic tattoos or biostamps are currently under development. They are rubber patches that have a layer of flexible silicon wires (Fig. 5). The idea is to create an electronic device that is thinner than a sheet of paper, as flexible as a Band-Aid , and that can stick to the skin [43]. Beside their ability to record and transmit vital signs, these sensors have the potential to measure electrolytes and lactates in skin fluids [44]. If reliable, they may bring further opportunities such as remote metabolic monitoring in patients with chronic disease.

### Toward home monitoring

During decades, cardiovascular monitoring belonged to high-level specialists working in specific locations such as catheterization laboratories, operating rooms and intensive care units. Today we enter into a new era where, thanks to wearable or implantable sensors, hemodynamic monitoring will become possible from home. It will create as many opportunities as it raises questions: Who should regulate the use of these new products and software applications, what and where is the frontier between medical and consumer products, can we trust the measurements, what should be monitored and in whom, who is going to receive, interpret and protect the information, what is the impact on patient care, and who is going to pay for this?

## Connectivity

Connectivity is the third transformation we can expect on a relatively short-term basis. It is a challenge for industry but a necessity for clinicians and patients. To quote Dr. Pronovost [45], director of the Armstrong Institute for Patient Safety and Quality at Johns Hopkins (Baltimore, MD, USA), “Imagine if Boeing were putting together an airliner and the maker of the landing gear were to say ‘we’re not going to send a signal to the cockpit to tell you if the landing gear is up or down, you’re going to have to guess’. Imagine if Boeing said ‘that will kill people and cost a lot of money, but if you don’t want to send the signal, don’t worry about it’. That is essentially what we, as health-care providers, are doing when we buy devices for the intensive care unit that can’t talk.” Some safety advantages of connectivity are obvious such as the communication between pumps delivering vasopressors and systems measuring arterial pressure. Cross-checking monitoring information coming from different sources (e.g., heart rate from EKG and pulse rate from pulse oximetry) may also decrease false alarms [46] or provide additional diagnostic information (e.g., the detection of electromechanical dissociation).

### Data integration

Beside its safety advantages, connectivity opens the door to data integration. First, it allows the automatic calculation of derived parameters such as oxygen delivery (requiring cardiac output, hemoglobin and arterial oxygen saturation at the same time) or transmural pulmonary artery occlusion pressure (requiring simultaneous pressure measurements from the pulmonary artery catheter and the mechanical ventilator), just to mention a few.

Second, data integration is the opportunity to build a more holistic representation for every patient. Hemodynamic parameters alone are usually insufficient to capture the whole clinical picture and hence select the optimum therapy. What are the comorbidities, what did the echocardiography show, are the lactates elevated, is the patient hypoxemic, does the patient receive inotropes or vasopressors, is he mechanically ventilated, is a PEEP applied? These are all basic questions part of the interpretation of hemodynamic variables. Integration of all monitored variables together with the patient’s history and laboratory tests coming from electronic medical record (EMR) systems opens the doors to the development of smart systems (artificial intelligence) able to suggest a diagnostic and/or a treatment, and even to deliver therapy (closed- or open-loop systems) [7]. Recent studies have investigated the feasibility of closed-loop systems to automatically administer fluid during the perioperative period [47]. As of today, such systems use only a few hemodynamic parameters as input variables. Their goal is to the decrease variability in fluid management during surgery, a large variability being associated with poor outcome [48]. Connectivity and data integration may allow the development of controllers able to process multiple parameters at the same time and hence guide or unload clinicians in more complex clinical situations.

### Predictive analytics

Data integration is also the cornerstone of predictive analytics. Predictive analytics are statistical methods (predictive modeling, machine learning, data mining) analyzing current and historical data to make predictions about the future. This is an intense area of research for experts in medical informatics. The complexity of predictive algorithms is limitless, but the principle is simple: The combination of several parameters (usually vital signs) and the automatic recognition of specific patterns allow the detection of adverse events, earlier than the classical side-by-side monitoring of single parameters [49]. Lockheed Martin, the American aerospace and defense leader, claims it is able to diagnose sepsis 14–16 h before doctors [50], by monitoring heart rate, respiratory rate, blood pressure and body temperature and using smart algorithms they initially developed to predict… the trajectory of missiles! Other algorithms have been developed to predict cardiorespiratory deterioration [51]. In this regard, predictive analytics may be useful to trigger the intervention of a rapid response team (RRT) and accelerate ICU admission (for patients in the ward or the emergency department) or to postpone ICU discharge for patients who are about to leave the unit. The simplification of monitoring systems, becoming smaller, smarter [52] and even wireless [53–55], should boost the clinical applicability of predictive analytics. Although a very exciting and promising research field, one have to acknowledge that these systems will never be able to predict the unpredictable, i.e., external interventions (vasoactive or inotropic drugs administration, mechanical ventilation) or accidents (surgical bleeding due to vessel injury) which are often the cause for changes in hemodynamic status and patient outcome.

## Conclusion

In 5–10 years from now, we can easily imagine a world where clinicians will learn hemodynamics with simulators and serious games and will monitor their patients with wearable or implantable sensors in the hospital and also after discharge. In the future, clinicians will use medical devices that communicate and integrate the clinical, physiologic and biological information necessary to predict adverse events, prevent medical errors, propose the most rationale therapy and ensure it is delivered properly. Many questions remain unanswered, and studies will have to demonstrate this in silico progress has clinical value and ideally is cost-effective. But considerable intellectual and financial investments are already made, from small and innovative start-ups to giants like Apple, Google and Microsoft, to ensure that some of these new ideas and products become soon a reality [56]. Welcome to the digital health era!

## Abbreviations

ICU: intensive care unit
EIT: electrical impedance tomography
PEEP: positive end-expiratory pressure
